# Malignant ureteral obstruction: Whether decompression really improves patient outcomes and quality of life?

**Published:** 2008

**Authors:** K. Muruganandham, Rakesh Kapoor

**Affiliations:** Department of Urology and Renal Transplantation, Sanjay Gandhi Post Graduate Institute of Medical Sciences, Lucknow - 226 014, Uttar Pradesh, India. E-mail: rkapoor@sgpgi.ac.in

## SUMMARY

Wong L *et al.*,[1] in a recent retrospective study of 102 (45 men, 57 women) patients who presented with malignant ureteric obstruction (MUO), analyzed outcome data including survival, complication rates and time spent in hospital following attempted decompression of urinary tract. They also identified the prognostic factors which predict an unfavorable outcome despite technical success of the procedure.

Median patient age at MUO diagnosis was 62 years. The source of primary malignancy was gastrointestinal in 21, gynecological in 32, urological in 30 and other sources (lymphoma, carcinoma breast etc) in 19 patients. At presentation 68% of patients had bilateral obstruction, 59% of patients had known metastases and 69% had symptoms of obstruction. Percutanoues nephrostomy(PCN) was the more common initial procedure (75%). There were 16 patients (16%) who had MUO at the first presentation of cancer whereas MUO developed in 86 patients (84%) with previously diagnosed cancer. The median follow-up of patients was 46 months (95% CI 32-108). Median overall survival (OS) was estimated at 6.8 months (95% CI 4.8- 9.3) and the OS rate at six and 12 months was estimated at 56% (95% CI 46%-65%) and 29% (95% CI 21%- 9%), respectively.

By univariate analysis, inferior OS was associated with presence of metastases (*P* = 0.041), prior therapy (*P* = 0.024), diagnosis of MUO in previously established malignancy (*P* = 0.043) and creatinine greater than 40 mmol/l (*P* = 0.088). Multivariate analysis revealed independent prognostic factors for inferior OS were presence of metastases (*P* = 0.020) and diagnosis of MUO in previously established malignancy (*P* = 0.039) Patients were divided into four risk groups according to the number of baseline unfavorable univariate OS prognostic factors. The OS differed significantly among the four risk groups (*P* = 0.011) with the 12-month OS rate ranging from 63% (95% CI 38-82%) for the cohort of patients having one or zero unfavorable factors to 12% (95% CI 3%- 8%) for the cohort of patients having all four.

A total of 54 patients (53%) had at least one complication associated with the initial or subsequent procedure. Significant complications occurred in two patients (2%) consisting of arterial hemorrhage and wound dehiscence. Infection and blockage were the most common complications. Of the 54 patients experiencing a complication, 16 (33%) had two or more than two complications. The complication rate was significantly higher for patients who had postoperative therapy for cancer (61% *vs.* 37% for no therapy, *P* = 0.03). There was no significant difference in complication rates between primary PCN (53%, 40 of 76) and retrograde stenting (RS) cases (56%, 14 of 25) (*P* = 0.82).

The overall median remaining lifetime spent in hospital was 17.4% (range 0.2-100%). Six patients spent all of their remaining life as hospital inpatients. Prolonged hospital stay was associated with patients who presented with symptoms (*P* = 0.012), who had no postoperative therapy for their cancer (*P* = 0.01) and had a PCN as the initial procedure (*P* = 0.043).[1]

## COMMENTS

The introduction of percutaneous techniques associated with the evolution of the field of endourology, the use of ultrasound and computerized tomography (CT) guidance and the routine use of perioperative antibiotics have dramatically reduced the morbidity of ureteral stent placement. Despite these technical advances in percutaneous and endoscopic procedures, there is little evidence in the literature to suggest improvement in patient outcomes and in particular, quality of life. Identification of adverse risk factors and the associated poor survival for patients will be helpful for clinicians in discussing prognosis and the benefit of MUO treatment with patients.

There are a few other retrospective studies reported in the literature addressing the issue of decompression in MUO and all have uniformly shown worse OS in this group of patients.[2–4]

In a prospective study involving 42 patients who underwent urinary decompression for malignant ureteric obstruction Harrington *et al.*, reported a median survival of 133 days and six months survival rate of 40% in spite of high initial procedural success.[5] Availability of further treatment still remains a commonly proposed reason for decompression.[2,3]

Since patients presenting with malignant ureteric obstruction usually have advanced malignancy the procedures used for urinary diversion are generally palliative. The palliative nature of these procedures makes the assessment of the quality of life (QOL) a very important objective factor to determine the success of urinary diversion in this scenario. Shekarriz B *et al.*, studied 103 patients with advanced malignancies who underwent palliative urinary diversion and analyzed performance status after diversion and survival for those patients. A modified Karnofsky performance scale (KPS) was used for assessment of physical performance. A scale of 0-4 was used: 0) hospitalized until death; 1) bedridden at home, severe pain despite analgesia; 2) moderate disability, moderate pain despite analgesia; 3) mild disability, pain-free with medication; and 4) normal. The median post-diversion KPS score was 2 (range, 0-4) and 15% of patients never left the hospital. Overall, 51% required secondary percutaneous procedures and 68.4% had complications (minor, 63%; major, 5.4%). Eighty-six per cent had cancer-related symptoms despite the diversion. The average survival was five months, 50% of which was spent in the hospital.[6]

Ideally, a randomized trial involving application of a formal QOL tool to patients with MUO having or not having decompression should be performed. However, it is difficult ethically to refuse decompression if a patient or the family requests it to be performed.

Decompression of the obstruction may merely prolong the patient's suffering. Patients who present with bilateral obstruction and uremia have a short life expectancy if their disease remains untreated. These patients appear to present in a more advanced stage of the disease. The functional status of these patients is generally poor at the time of presentation secondary to preexisting conditions related to their malignancy. Even with the recent advances in the field of endourology, the QOL and survival of patients with malignant ureteral obstruction remains poor. The metastatic status of the primary disease and availability of treatment modality should be assessed in discussion with other specialty colleagues, especially for non-urological malignancies before decompression. Patients and family members should be informed of the poor outcomes after palliative procedures to guide them toward appropriate decision-making. Decompression for malignant ureteral obstruction should not be a knee-jerk response. It should only be pursued after careful assessment of unfavorable factors and thoughtful counseling.
